# Robotic Motion Learning Framework to Promote Social Engagement

**DOI:** 10.3390/app8020241

**Published:** 2018-02-05

**Authors:** Rachael Burns, Myounghoon Jeon, Chung Hyuk Park

**Affiliations:** 1 The George Washington University;; 2 Michigan Technological University;

**Keywords:** human-robot interaction, socially assistive robotics, imitation, motion learning

## Abstract

Imitation is a powerful component of communication between people, and it poses an important implication in improving the quality of interaction in the field of human-robot interaction (HRI). This paper discusses a novel framework designed to improve human-robot interaction through robotic imitation of a participant’s gestures. In our experiment, a humanoid robotic agent socializes with and plays games with a participant. For the experimental group, the robot additionally imitates one of the participant’s novel gestures during a play session. We hypothesize that the robot’s use of imitation will increase the participant’s openness towards engaging with the robot. Experimental results from a user study of 12 subjects show that post-imitation, experimental subjects displayed a more positive emotional state, had higher instances of mood contagion towards the robot, and interpreted the robot to have a higher level of autonomy than their control group counterparts did. These results point to an increased participant interest in engagement fueled by personalized imitation during interaction.

## Featured Application

Robotic imitation during human-robot interaction can build interest, empathy, and trust in human participants, opening the door to a vast host of medical care applications. These include a safe and realistic platform for special needs children to practice social encounters, companionship for elderly or bedridden hospital patients, and reliable supervision of outpatients.

## 1. Introduction

### 1.1. Imitation in Humans

Imitation is a social response ingrained in human behavior, which serves to promote group assimilation and empathy. The “Chameleon Effect” refers to the phenomenon where humans unintentionally mimic the behaviors of another person as they interact. Additionally, the recipient of this imitation passively develops empathy and rapport for the interaction participant [1]. The Chameleon Effect is responsible for both gestural imitation (“motor contagion”) and emotional imitation (“mood contagion”) [2].

Along with promoting subconscious adaptation to a social group environment, imitation is a prominent tool for conscious neurological development. “Mirroring” refers to the learning method prominent in early childhood development, where specific “mirror neurons” enable young children to develop behavioral, language and motor skills through observation [3]. The benefits of mirroring are two-fold: the child develops crucial functional skills and a sense of belonging by imitating peers in their group.

While imitation and social engagement are innate behaviors for neurotypical people, these skills can be very difficult for people with neurological conditions. Autism Spectrum Disorder (ASD) is a neurodevelopmental condition that now affects 1 in 68 children in the United States, according to the US Center for Disease Control (CDC). The Diagnostic and Statistical Manual of Mental Disorders – Fifth Edition (DSM-5) defines this condition as “persistent difficulties in the social use of verbal and nonverbal communication…[resulting] in functional limitations in effective communication, social participation, social relationships…individually or in combination”.

Children with ASD may differ from neurotypical children in areas such as sensory interpretation, communication ability, and emotional response. Additionally, mirror neurons in children with ASD have been found to not always function, resulting in a diminished ability to learn social behaviors through mirroring [3]. These compounding factors can make it difficult for children with ASD to interact with their peers, leading to feelings of frustration and isolation. These children need a safe environment to practice and hone their social skills without fear of ridicule.

### 1.2. Imitation in Robots

Human-robot interaction (HRI) may serve as a “bridge” in social interaction for people with neurological disorders. Robotic therapy continues to grow with interest as a viable method for increasing social engagement skills in children with ASD. Studies have shown that children with ASD find robots to be more approachable than human strangers [4]. Additionally, children paired with robot mediators in therapy showed an increase in shared attention and facial expression imitation of the mediator, in comparison to children paired with a human mediator [4]. Overall, research suggests that children with ASD find robots more interesting and engaging than human therapists, improving interaction in therapy sessions [5].

Neurotypical children enjoy interacting with robots as well, provided that the robot’s behavior stays within parameters similar to standard human-human interaction. Users prefer robots to stand at a moderate distance (at a “social” distance, versus “personal” or “intimate”), provide moderate eye contact, and not wait an extended period of time to respond to the user [6]. Finally, people prefer interacting with a robot that appears to provide adaptive and intelligent responses [7].

Evidence of humans displaying the Chameleon Effect toward robots has been documented in multiple studies. In [8], participants were paired with either a human or robot demonstrator. The user was asked to copy the demonstrator and relocate their empty hand or a held object to the matching end position. The manner in which they reached the end goal was not specified. However, the results showed that the participant tended to imitate the trajectory and velocity with which the demonstrator reached the end goal, indicating motor contagion. In [9], participants were asked to use a Nintendo Wii remote to mime gestures simultaneously with a robot. Participants noted feeling “more comfortable” while completing the task when the robot matched their gesture speed, rather than moving at its preset pace. Change in the robot’s motor behavior to match the user’s speed invoked an empathetic Chameleon Effect response and improved the participants’ overall perception of the robot. In [2], users played a pose imitation game with NAO, a small humanoid robot. NAO demonstrated increasingly complex gestures with either a positive or negative mood. The researchers found that, during the easy version of the game, users were likely to display mood contagion and match the robot’s demeanor along with the poses. These studies indicate that robotic imitation encourages humans to perceive robots as equivalent social agents when working together in a goal-oriented setting.

This research aims to determine the effect of robotic imitation on participant behavior during a structured social setting, rather than a goal-oriented setting. In this study, participants engage in a two-part game with ROBOTIS-OP2, a small, humanoid robot. The participant and robot take turns demonstrating unique gestures, but unlike the above studies, the two agents do not work together to complete an official task. The experimenter aims to determine whether robotic imitation will elicit increased user engagement during a 1:1 human-robot social interaction, instead of the human and robot completing a physical, task-oriented interaction.

## 2. System Design and Algorithms

### 2.1. Robotic Learning from Gestures

The robot was programmed to imitate human gestures by implementing a motion learning algorithm called “Dynamic movement primitive with Gaussian mixture regression” (DMP with GMR). A dynamic movement primitive (DMP) is a generalized gesture with specific position goals and end points joined in a sequence to create a scalable movement based on an original demonstration. Robots utilize DMPs to reproduce core movements in variable settings - for example, when the distance or pathway between the start and end goal changes. This is also referred to as “motion learning”.

(1) $$\left[\begin{array}{c} \dot{z}\\ \dot{y}\\ \dot{x} \end{array}\right]=\left[\begin{array}{c} \alpha \left(\beta \left(y^{g}-y\right)-z\right)+x*f\left(x\right))/\tau \\ z/\tau \\ -\alpha_{x}x/\tau \end{array}\right]\text{ with initial state }\left[\begin{array}{c} 0\\ y_{0}\\ 1 \end{array}\right]\text{ and attractor state }\left[\begin{array}{c} 0\\ y^{g}\\ 0 \end{array}\right],$$

Eq. 1 is the basis from which all motion learning controls are developed [10]. It is a PD control signal, separated into three first order equations, modified to produce a custom trajectory. *α*(*β*(*y*^*g*^ − *y*) − *z*) is a spring damper system, where y refers to the system state, *y*^*g*^ refers to the goal state, and *z* refers to the velocity of the system. The constant variables α and β are gains, and τ represents temporal scaling. A τ value between 0 and 1 slows the reproduction rate, whereas a τ value greater than 1 increases the speed. The forcing function f(x) allows the algorithm to create a specific trajectory, rather than taking the shortest path from the start to end goals. This is multiplied by x, the diminishing gate, which restrains the boundaries of the trajectory in order to reach the proper end goal [11]. Eq. 1 determines a DMP in one dimension. To generate multi-dimensional movement reproductions, the equation’s forcing function is made phase dependent instead of time dependent and is run multiple times with concurrent phases.

**Figure 1.**
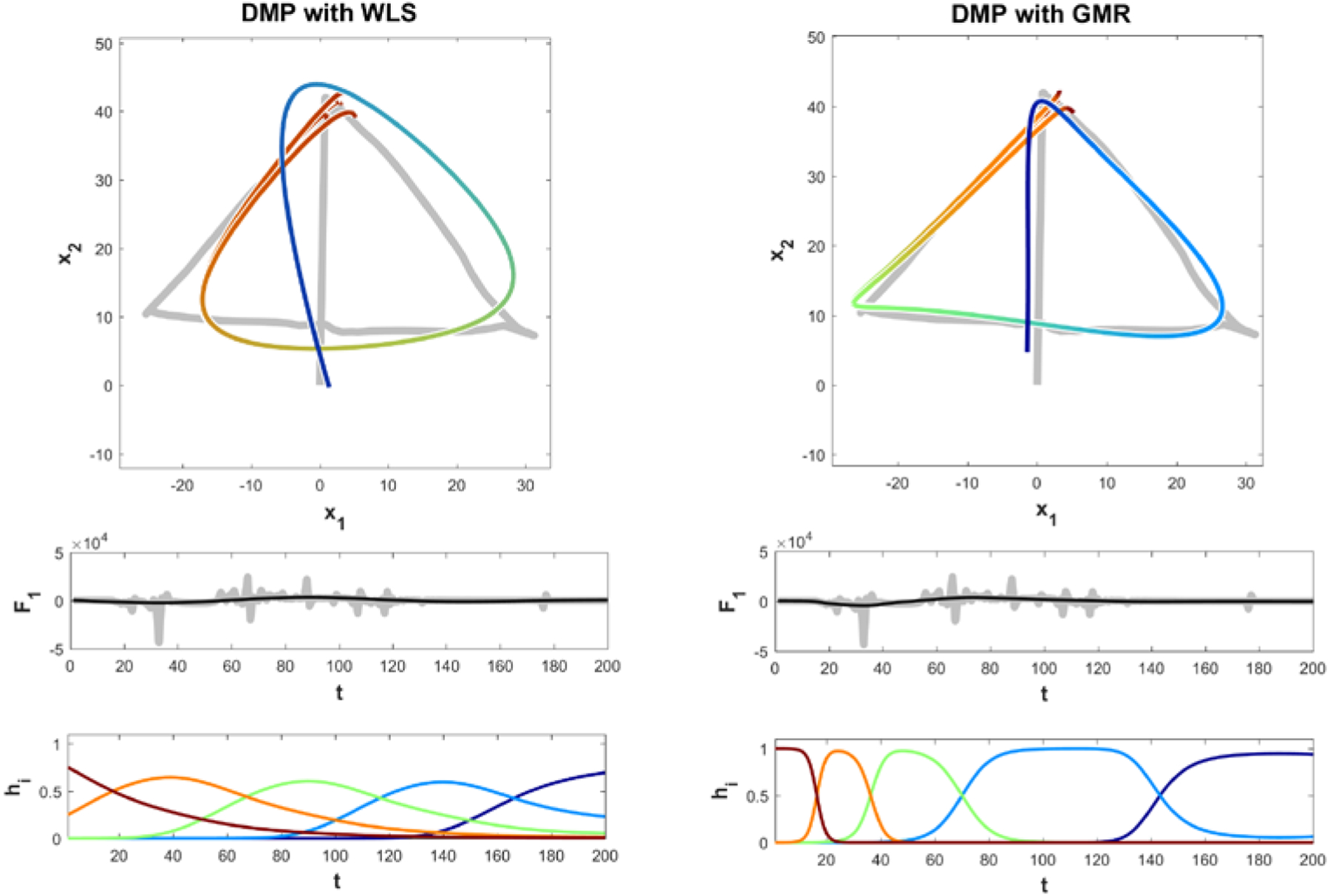
Top: A comparison of the reproduction accuracy of two motion learning systems (the rainbow overlays) for a two dimensional drawing (underneath, in gray). Middle: Grey bars represent the force components exerted when creating the original trajectory over time. Note that the forces are strongest when the trajectory forms each corner of the triangle. The activation states of GMR are automatically assigned based on the perturbation of the force on the system’s replication of the original trajectory. Bottom: The five activation states of each motion learning algorithm. WLS activation states fire after equal intervals of time, whereas GMR activation states fire at corners, which are the defining portions of this particular trajectory.

Multiple variations of motion learning algorithms were compared to determine which would be appropriate for humanoid robotic movements. Most differences in motion learning algorithms lie in how the forcing function is calculated. Forcing functions redirect the trajectory into specific movement reproductions through activation states, also referred to as activation weights. For standard DMP techniques, such as DMP with weighted least squares (WLS), the duration, position, and intensity of the activation weights are preset equidistant throughout the execution of the movement reproduction. For DMP with Gaussian mixture regression (GMR), rather than an equal distribution, the activation states are placed at key points that define the trajectory, which the algorithm identifies during learning [11]. The results of our comparison of WLS and GMR learning algorithms can be seen in Fig. 1.

The forcing function in DMP with GMR causes the algorithm to have more precise movement reproductions than traditional methods. The forcing function is calculated using a series of statistical models. First, a Gaussian Mixture Model (GMM) plots the raw biological motion mathematically, as “data points generated from a mixture of a finite number of Gaussian distributions with unknown parameters” [12]. Next, an expectation maximization (EM) procedure extracts important features of the GMM, including mixing coefficients, centers, and covariances of the system, to estimate the boundaries of the reproduction trajectory [13]. This builds the trajectory. Finally, a Gaussian Mixture Regression (GMR) is implemented. This determines the velocity and acceleration with which the trajectory is traversed. GMR is used for each iteration of the system to calculate whether the magnitude of the current activation state is sufficient to maintain the desired trajectory, or whether the system must utilize the next activation state. Due to its superior results during our preliminary comparisons, DMP with GMR was chosen as the motion learning algorithm for this study.

This research brings multiple contributions to the DMP with GMR algorithm. Firstly, it adds angle-based reproduction. The original open-source DMP with GMR code solely performed point-based reproduction. In point-based reproduction, the goal state is a set of specified coordinates in three-dimensional space. The physical arrangement and angles of the robot limbs used to achieve this point are irrelevant. This can lead to unnatural limb positioning. In angle-based reproduction, the goal is for the robot’s end effector angles to match the specified angles, resulting in a pose that mimics the user’s own. We developed the code to produce angle-based replication by modifying the program inputs. The DMP with GMR algorithm plots two inputs, and then attempts to recreate the resulting line graph. Substituting angle inputs for Cartesian coordinate values resulted in angle-based reproduction values, rather than point-based ones.

**Figure 2.**
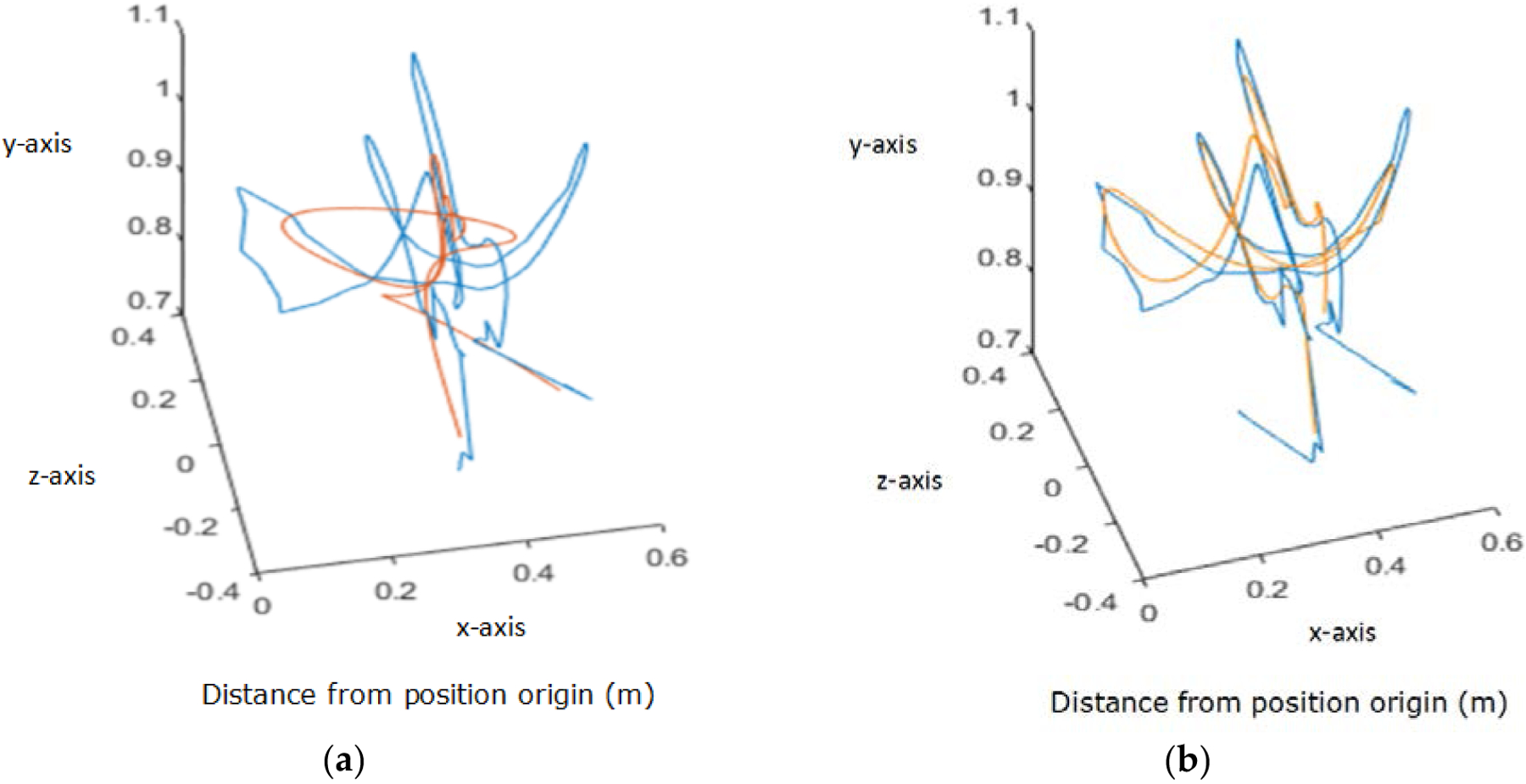
In blue, the original 3D trajectory of a participant’s right hand as they perform a “conducting” gesture, tracked using a Microsoft Kinect. *Left*: Motion learning reproduction without segmentation and fusion. *Right*: Reproduction after trajectory segmentation and fusion, using 90 point segments with a 20 point overlap.

This research improved the reproduction accuracy of the algorithm by incorporating trajectory segmentation and fusion. A single user demonstration may contain multiple unique gestures, and therefore contain multiple movement primitives. However, the more primitives, or points within a primitive, are assigned to an activation state, the less accurate the reproduction will be. Additionally, there is not a linear relationship between the number of activation states implemented and the amount of time needed to run the algorithm – it takes longer to run one long segment with 20 activation states than it does to run four short segments with 5 activation states each. Therefore, in order to create the most accurate and time efficient reproduction, a trajectory should be separated into primitives before being processed by the motion learning algorithm. The segments are then rejoined before imitation, resulting in one full-length, higher accuracy reproduction. An example of this improvement to the methodology can be seen below in Figure 2. The blue original trajectory is roughly 350 points long. On the left is the reproduction (overlaid in orange), created using the motion learning algorithm once on the entire length, using ten activation states. On the right is the reproduction created using the motion learning algorithm on 90 point segments, with a 20 point overlap between segments, also using ten activation states.

Finally, dynamic modulation of the activation state input was added to the DMP with GMR algorithm. In DMP with GMR, activation states are used to map the most drastic changes in system trajectory. The number of activation states needed vary based on the complexity of the trajectory segment presented. If too few states are used, the DMP recreation will be oversimplified beyond recognition. If too many states are used, the system will crash because it has run out of places to utilize the states. Therefore, it is imperative that the motion learning algorithm is equipped with a forcing function that can dynamically modify the number of activation states used for each new trajectory segment. A program was created that adjusts the number of activation states used in recreating a gesture, based on the accuracy of the reproduction. The percent error threshold may be modified by the user. If the reproduction is above the specified percent error, then the motion learning program is rerun with an increased number of activation states. This process is repeated until either the reproduction is within the specified percent error or the number of activation states is 25. This was determined to be the highest number of states that could be utilized before crashing the system.

### 2.2. Robotic Platform

The robotic system chosen for this experiment was ROBOTIS-OP2 (“OP2”), a humanoid robot standing at 17.9 inches (45.45 cm) and weighing 25 pounds. OP2 has PID controllers at 20 different joints, can talk through Text-to-Speech or pre-recorded audio files, and is programmable directly through a Linux OS. OP2 was chosen as a suitable robotic agent for this experiment due to its small size, friendly humanoid appearance, multimodal feedback source capabilities and computer-sized processing power.

**Figure 3.**
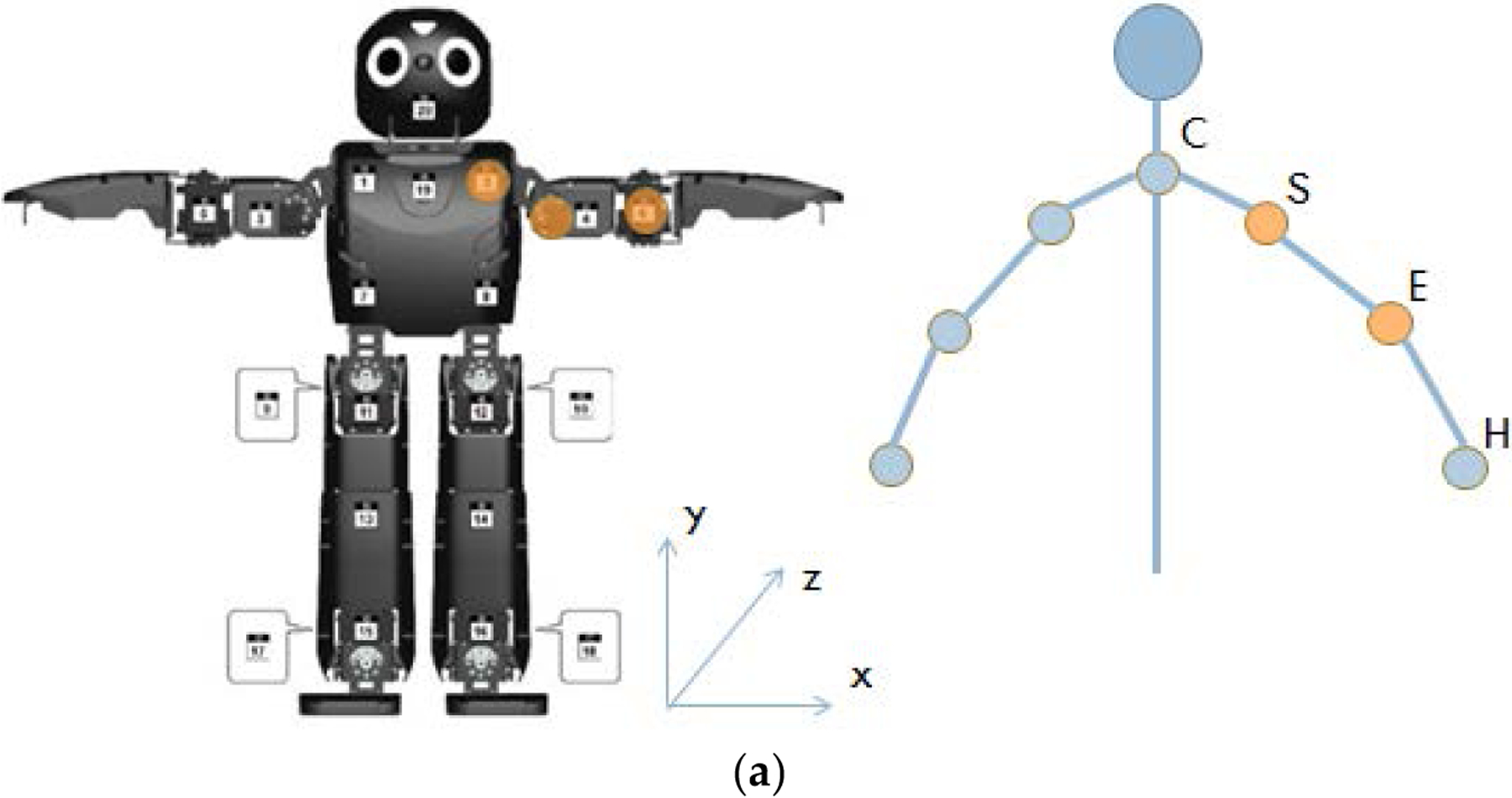
A diagram of OP2 on the left, in comparison to a person on the right, where C stands for center, S for shoulder, E for elbow, and H for hand. OP2 is capable of reproducing the 2 degrees of freedom (DoF) found in the human shoulder and the 1 DoF in a human elbow, but cannot reproduce hand or wrist movement.

For this experiment, the motion learning reproduction was implemented only in OP2’s arms (shoulders and elbows), as seen in Fig. 3. Reproduction of torso, hip, or leg movement would require additional programming for stabilization, as OP2 does not have the same bodily proportions as a human, and additionally differs in terms of weight distribution. OP2 has two motors in each shoulder. These motors can be set to replicate human shoulder pitch and roll. Manipulating these two planes allows arm yaw to be replicated as well. OP2 has one motor in each elbow, enabling it to match human elbow pitch. However, OP2 lacks the ability to perform the rotational movement of wrist yaw. This means that it cannot reproduce the human movement of rotating the palm from facedown to faceup. However, by replicating the angles of each participant’s shoulder and elbows, the majority of the gesture was able to be articulated. Perhaps because they saw that OP2 had no hands, participants tended to close their fists or generally not flex their hands when demonstrating movements to the robot.

The Microsoft Kinect, a depth camera, was used to track participant movement. A custom GUI was developed to utilize depth information from the Kinect to generate a skeletal framework of the participant’s joints for movement reproduction by OP2. Arm joint information was collected and used by the GUI to calculate arm angles. Eight angles were tracked in total – the four left arm angles listed in Table 1, and their right-side equivalents. These angle variables became inputs into the DMP with GMR motion learning algorithm in MATLAB. New, smoothed, similar angle reproductions were generated by the algorithm from the demonstration data. These values were then converted from degrees into motor actuator positions, which would allow for OP2 to be controlled. The values were outputted to a text file, remotely uploaded into OP2’s directory, and called from our “Emotion Game” program to perform gesture reproduction on command.

The aim of this experiment was to determine whether imitation increased social engagement during human-robot interaction. In order to test this hypothesis, a social situation that prompted interaction first had to be constructed. Therefore, a set of 12 emotion primitives was developed for OP2. Our emotions were chosen based on Russell’s Circumplex Model of Affect. This model breaks emotions down into two axes: arousal, which refers to the energy level present with the emotion, and valence, which indicates whether the emotion is a positive or negative experience. Between these two axes, all levels of emotion can be represented [14]. The 12 primitives were carefully chosen to display a full range of the emotional spectrum and maximize the chance of eliciting responses from the participant. The robot demonstrated the emotions to participants in the following order: sadness, fear, excitement, disgust, curiosity, pride, anger, embarrassment, surprise, pleasure, frustration, and tiredness. Descriptions of the gesture and phrases used to demonstrate these emotions can be found in Table A1.

Twelve participants were recruited for the study; 5 female and 7 male, ages 19–30 (M = 23, SD = 3.55). Two participants self-reported as Asian; 2 as Hispanic, and 8 as Caucasian. All participants self-identified as neurotypically functioning adults. The participants were 6 graduate students and 6 undergraduate students in STEM subjects. In a user survey, all participants selected either “Agree” or “Strongly Agree” when asked if they considered themselves proficient in “current” technology, such as mobile devices. When prompted whether they had prior experience with robots, the majority of participants chose “Agree” or “Strongly Agree”, with 3 participants instead choosing “Disagree”. All participants were randomly assigned into Control or Experimental groups. The experimental protocol was reviewed and approved by the George Washington University Office of Human Research (OHR) Internal Review Board (IRB) under the IRB Protocol Number 111540. All participants were given a packet containing project IRB approval and their informed consent information prior to data collection.

### 2.3. Experimental Setup

**Figure 4.**
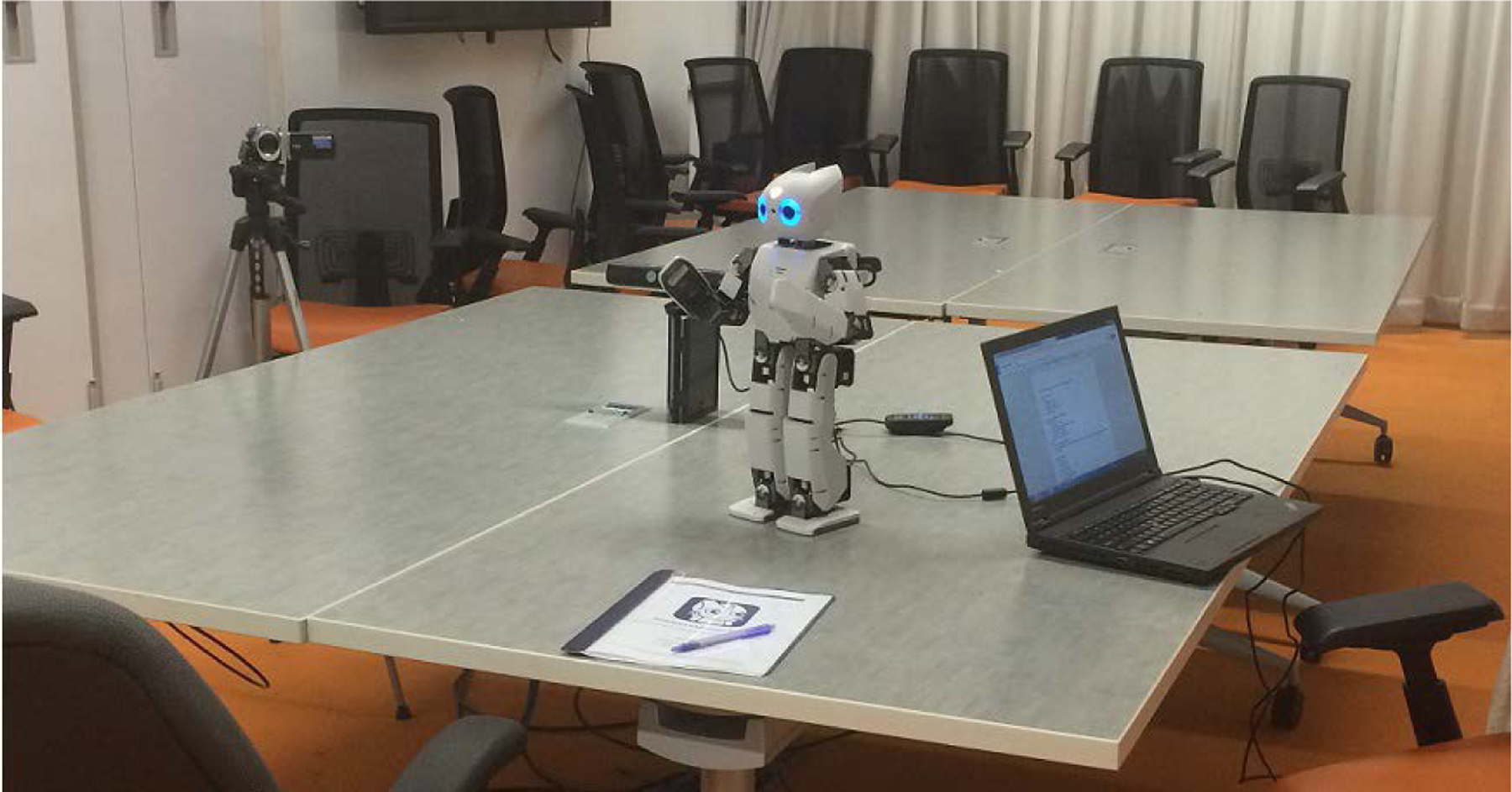
The experiment setup, consisting of a video camera (left), Kinect (behind robot), OP2, a laptop to operate OP2, and the informed consent packet.

For the experiment, each participant was directed to an unoccupied room containing a conference table equipped with a video camera, Microsoft Kinect, laptop, OP2, and seating for at least two people. The cords to power the equipment were hidden underneath the table to minimize participant distraction. The experimenter sat adjacent to the participant, with OP2 placed in between them on the table. The Kinect system was initialized and videotaping started. Participants engaged in a two-part “Emotion Game” with OP2. This layout can be seen below, in Figure 4.

In the first half of the Emotion Game, OP2 introduced itself, using pre-recorded audio and gestures triggered by the experimenter. The participant was prompted by OP2 to perform three gestures: one “exercise” move, one “emotion”, and one “dance” move. To ensure accurate data collection, each participant was asked to stand when demonstrating his or her moves. The angles of the participant’s limbs during these gestures were tracked and recorded by the Kinect. Video footage of each participant’s gestures was recorded to later be compared to the robot’s reenactments. The participant was offered to return to a sitting position after completing the demonstrations.

An interim-survey was completed by each participant after the first half of the emotion game. For each of its three sections, the participant was asked to answer Likert scale questions by assigning a numerical response to each prompt. The numerical scale ranged from 1–5, where “1” equated to “Strongly Disagree” and “5” equated to “Strongly Agree”.

The first section of the interim-survey examined the participant’s interest in and comfort with current and new technology. This was intended to help the experimenter see if self-proclaimed technological expertise and exposure would affect how a participant reacted to OP2. The second section prompted the participant to comment on their initial impression of the robot. These 5 Likert scale questions guided the participant to comment on the appeal of the robot’s demeanor, voice, word choice, and fluidity of movement, as well as whether he or she found the robot intimidating, to identify whether any of these factors were negatively impacting the participant’s interaction. The third section of the interim-survey and the sole section of the forthcoming post-survey both focused on the participant’s interaction with OP2, before and after the second half of the emotion game. The statements prompted the participant about his or her levels of comfort and enjoyment while interacting with the robot. The brief time required to complete the interim-survey allowed the experimenter to seamlessly train the motion learning algorithm for experimental group participants.

Each participant then completed the second half of the Emotion Game. OP2 announced to the participant that it would reciprocate by demonstrating gestures to them. The participant’s job was to identify which emotion the robot was attempting to articulate and display. The order of emotion primitives, listed earlier, appeared random but was kept consistent for all participants. However, OP2 performed an additional “DMP move” after the “curiosity” emotion primitive for experimental group participants. This “DMP move” was a DMP-learned reproduction of one of the participant’s previous gestures from the first half. Experimental group participants were not given forewarning that the robot would include the imitative gesture. The participants’ movements were tracked by the Kinect for posture analysis. Video footage was collected to track changes in emotional state, including mood contagion.

Four dependent variables were tracked to determine where imitation increased social engagement during human-robot interaction: participant posture, facial emotional state, instances of mood contagion, and perception of robot autonomy.

For posture analysis, an algorithm was used to quantify the participants’ interest in OP2 through bodily movement and posture. Using the Kinect skeletal framework GUI, four points on the participant’s body – the left and right shoulders and hands – were digitally connected to form a trapezoid. A large trapezoid area indicated that the participant’s limbs were spaced farther apart, indicating open body posture by leaning back and sitting upright. A smaller area indicated the participant’s limbs were closer together, suggesting the participant was slouching forward. Sitting upright is an indication of engagement, whereas slouching forward indicates the participant is withdrawn and disinterested in the robot’s performance [15]. The trapezoid areas before and after imitation were compared.

For facial emotional state, the user’s facial reactions to the various emotion primitives of the second half of the game were tracked using video footage. Smiling is a universal way to signal a positive emotional state, or a response to a particularly pleasing interaction. Unique instances of emoting, separate from the participant’s neutral expression, were recorded.

Mood contagion occurs when a participant unwittingly switches their mood to match or complement that of the opposing social agent. Instances of mood contagion provide us with another way to quantify the user’s perception of the robot as a social agent, as well as their engagement in the emotion game. Episodes of mood contagion were tracked using video footage.

Participants’ perception of robot autonomy was tracked using the results of the interim and post surveys – in particular, questions 3.3, 4.4, and 4.6. These questions were, in order, “The robot pays attention to me” (pre-imitation), “The robot was interacting with me” (post-imitation), and “The robot was paying attention to me” (post-imitation). While OP2 was controlled by the experimenter for this study, it was important to determine whether the robot maintained an appearance of autonomy. By recording the participants’ responses to these questions in particular, we can observe how incorporating the imitation gesture into their interaction improved or reduced the participants’ perception of the robot’s level of autonomy.

## 3. Results

### 3.1. Posture Analysis Results

**Figure 5.**
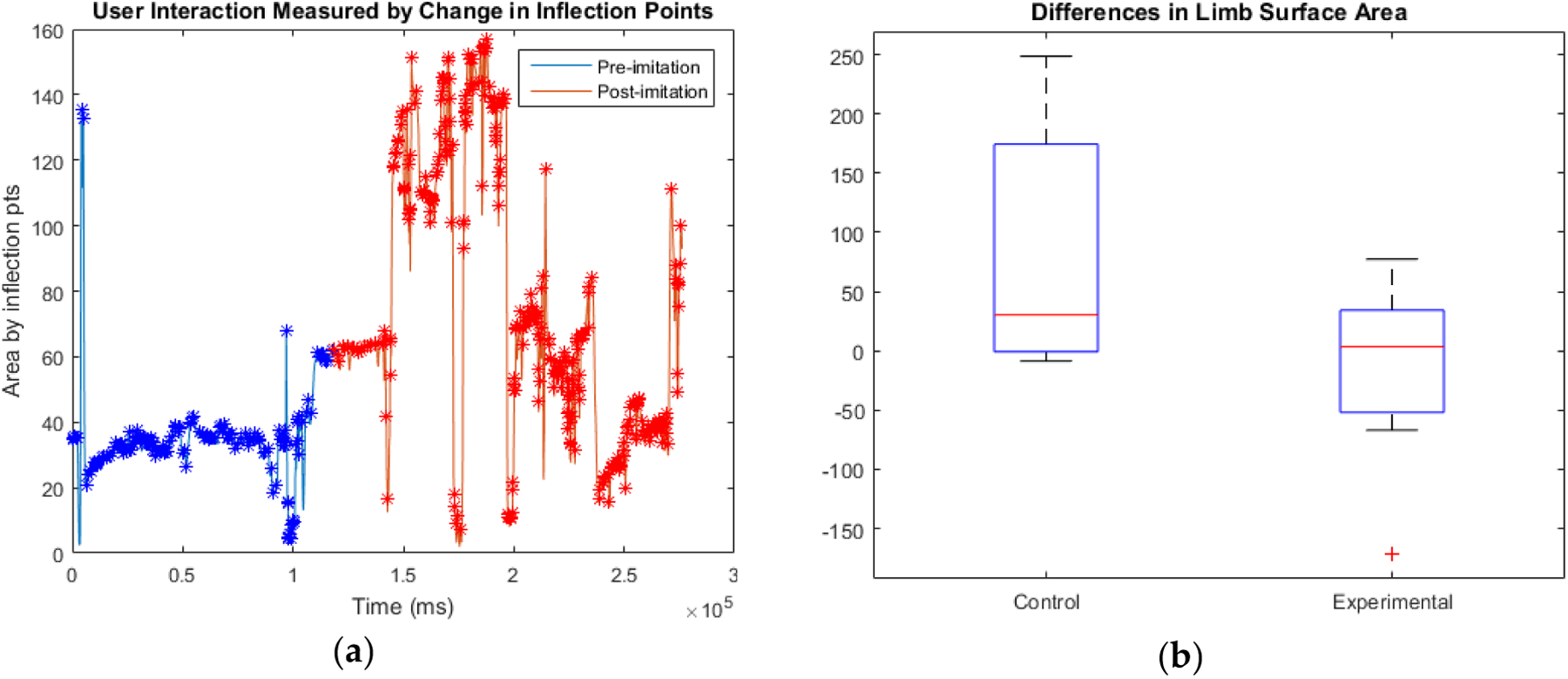
(**a**) The posture tracking of a participant who responded very noticeably to the imitative gesture with an increase in average body surface area. The imitation time stamp is indicated by a switch from blue to red markers; (**b**) Box-and-whisker plot comparisons of the change in surface area of participant posture for control and experimental participants.

The posture analysis data were separated into before and after imitation. This was visually represented by a change in color, as seen in Fig. 5a. Posture data was recorded at 30fps. Posture data file length ranged from 5,300–10,500 sample area points, depending on how quickly the participant guessed each emotion primitive. Area points that were local extrema were referred to as “inflection points”. Inflection points were calculated using a moving average window with a range of 15 samples. After all inflection points were calculated, average “before imitation” (A1) and “after imitation” (A2) inflection points were calculated. This was done by averaging every ten inflection points, and then finding the final mean of all those averages. In control participants, where imitation was not performed, A1 and A2 were calculated using the time checkpoint where the imitation would have occurred as the divider. The A1, A2, and ΔA of each participant can be seen in Table A2, in the Appendix. The mean ΔA of each group, as well as our statistical calculations, can be seen below in Table 2.

Four control participants had increased posture surface area from beginning to end of the emotion game, without imitation input. Only one experienced a decrease in the surface area. Four of the experimental group participants showed an increased average surface area after viewing the imitation gesture. Three demonstrated a decrease in surface area – one of these results was determined to be an outlier due to the extremity of the change.

A paired samples t-test was used to separately evaluate the changes in the surface area for the control and experimental groups. The control group t-value was found to be 1.694 for 4df, giving p = 0.165515. The experimental group t-value, including the outlier, was −0.53 for 6df, giving p = 0.615143. Therefore, neither group displayed a significant change in posture behavior. This result shows that the robot’s sudden intervention with imitation did not negatively affect the interaction.

### 3.2. Facial Measurement of Emotional State Results

**Figure 6.**
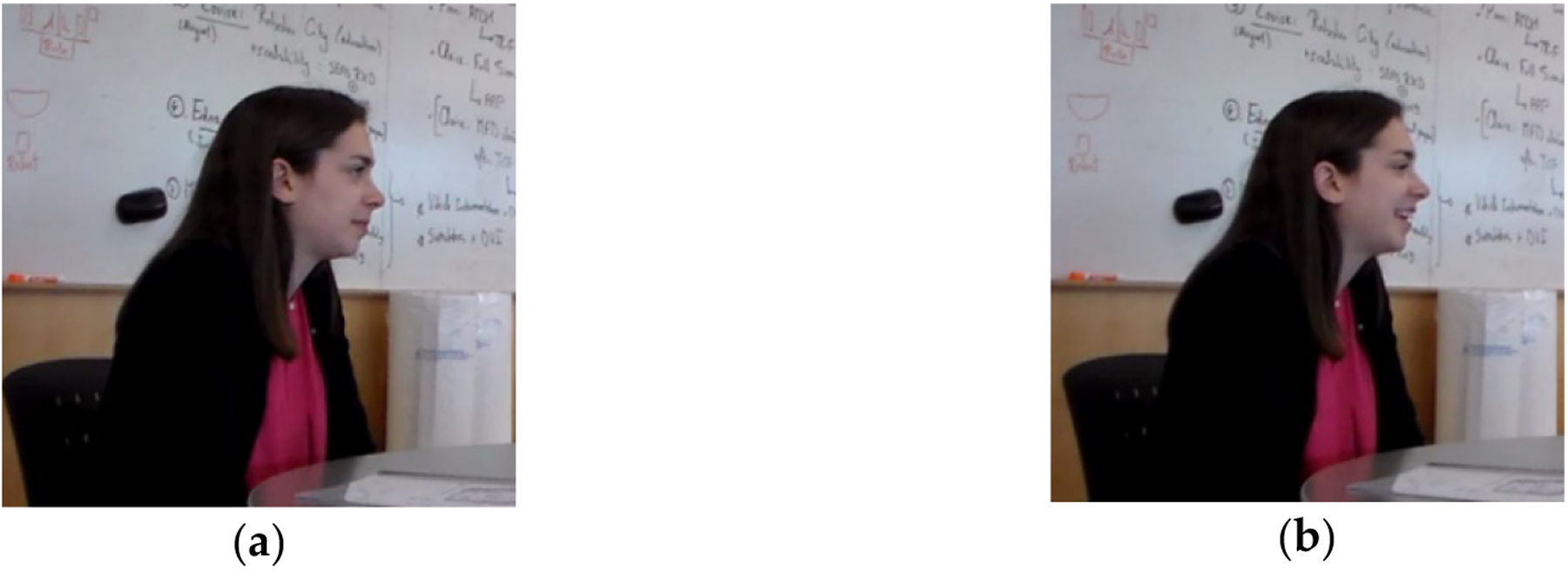
Comparison between (a) a participant’s neutral expression and (b) a positive facial expression.

The researchers observed the rate with which participants responded positively to viewing new robot emotion primitives, as in Fig 6. For experimental group participants, the rate of positive facial expression emoting was compared before and after witnessing the imitation gesture. For control participants, the positive emoting rate was compared before and after the corresponding time stamp.

**Figure 7.**
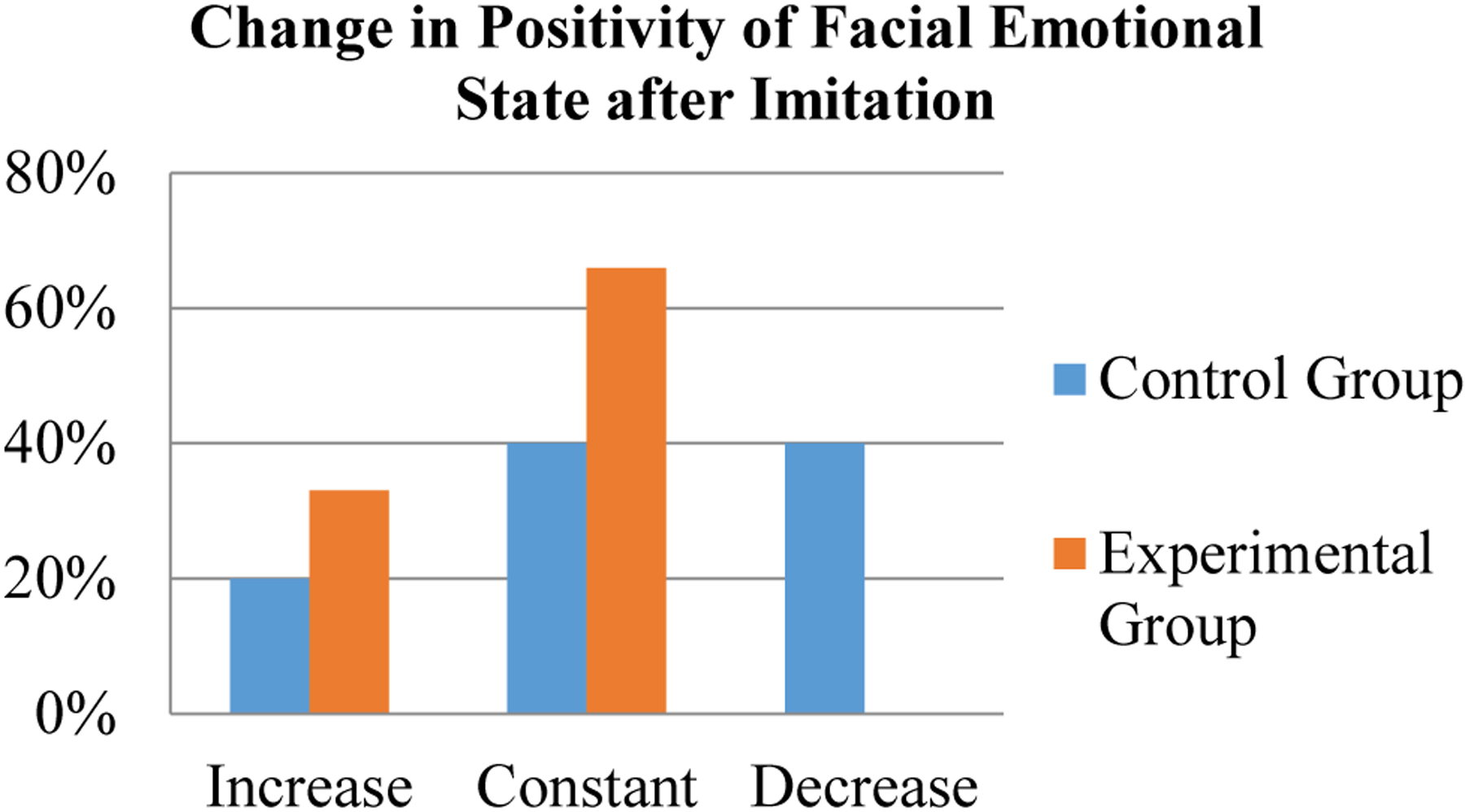
Bar graph comparison of the change in facial emotional state of control and experimental groups after imitation. Participants in the experimental group emoted with the same or increased positivity after imitation. The control group had mixed responses, including a decrease in positivity.

For the control group, two participants displayed a decrease in the rate of positive facial emoting, two maintained the same rate, and one increased. For the experimental group, two participants displayed an increase in positive emoting, and four stayed constant. One experimental participant’s rate could not be established due to an error in the video recording. A graphical representation can be seen below, in Fig. 7. A full comparison of participants’ emoting before and after imitation can be seen in Table A3, in Appendix A.

An unpaired t-test was performed to compare rate of positive facial expressions demonstrated by both the control and experimental groups. This can be seen below, in Table 3. Due to the low number of participants, the data was found to not be statistically significant, with a p-value of 0.46.

It is important to note that a smile is not always reflective of a positive emotional state. Humans can also demonstrate fake smiles in uncomfortable situations. To verify whether the participants genuinely enjoyed their experiences playing with OP2, and whether our facial emotional data was truly reflective of their experiences, we reviewed their survey answers. In particular, three questions were analyzed: Q3.4 from the interim survey, and Q4.1 and Q4.5 from the post-survey. The full prompts can be seen below in Table 4. The entire survey, as well as all participants’ individual answers, can be found in Appendix B.

The Likert scale answers from the control group participants all either increased in positivity or stayed the same between the interim- and post-survey. The average score for Q3.4 for the control group was a 4, which corresponds exactly with the Likert scale choice “I Agree”. The average of Q4.1 and 4.5 for the control group was a 4.3. From this information, we can infer that the control participants’ smiles were genuine, and the facial emotional state data for the control group is valid.

The Likert scale answers for 5 of the 7 experimental group participants either increased in positivity or stayed the same between the interim- and post-survey. Of the two experimental group participants who stated a decrease in enjoyment, one was Participant G, whose video data was unavailable and therefore had no emotion rate data. The other participant who had decreased enjoyment had an emotion rate that stayed constant. Therefore, one of the six experimental group participants with emotion rate data may have had some instances of false smiles. However, the average score for Q3.4 for the experimental group was a 4.57, and the average of Q4.1 and 4.5 for the control group was a 4.64. From this information, we can infer that the majority of the experimental group participants’ smiles were genuine, and the facial emotional state data for the experimental group is valid.

### 3.3. Mood Contagion Results

**Figure 8.**
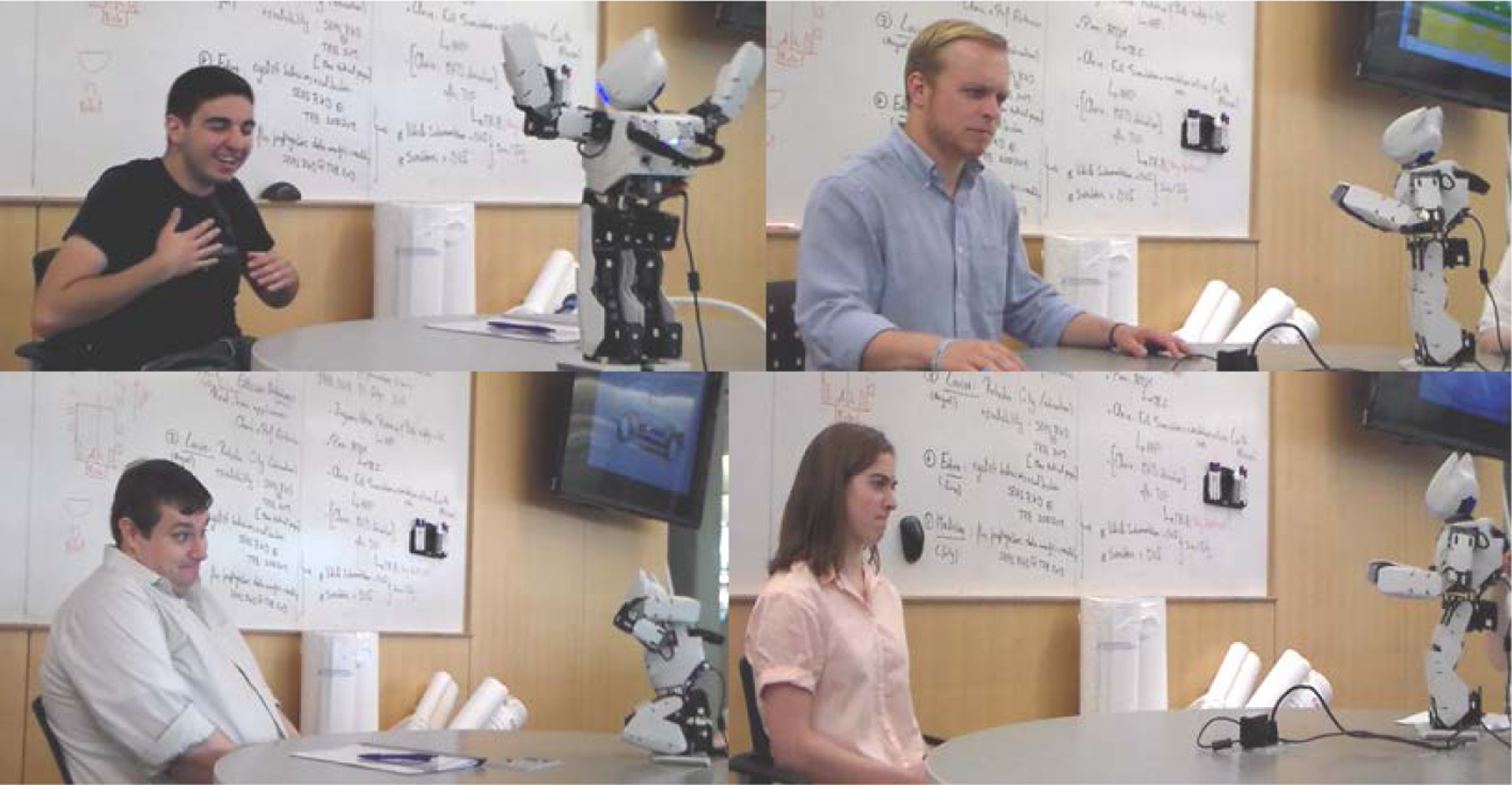
Participants displaying mood contagion towards various OP2 emotion primitives.

Of the twelve participants in this study, seven participants exhibited mood contagion - two from the control group, and five from the experimental group. Therefore, mood contagion analysis was only performed for these seven participants. Examples of mood contagion during the experiment can be seen above, in Fig. 8. Only two of the five experimental participants showed mood contagion before and after the DMP move. Two experimental participants only emoted before the imitation. The video recording cut off for the fifth participant, and so it is unclear if Participant G continued to display mood contagion after the imitation event. The emotion primitives that caused mood contagion can be seen below, in Table 5.

### 3.4. Participants’ Perception of Robot Autonomy Results

**Figure 9.**
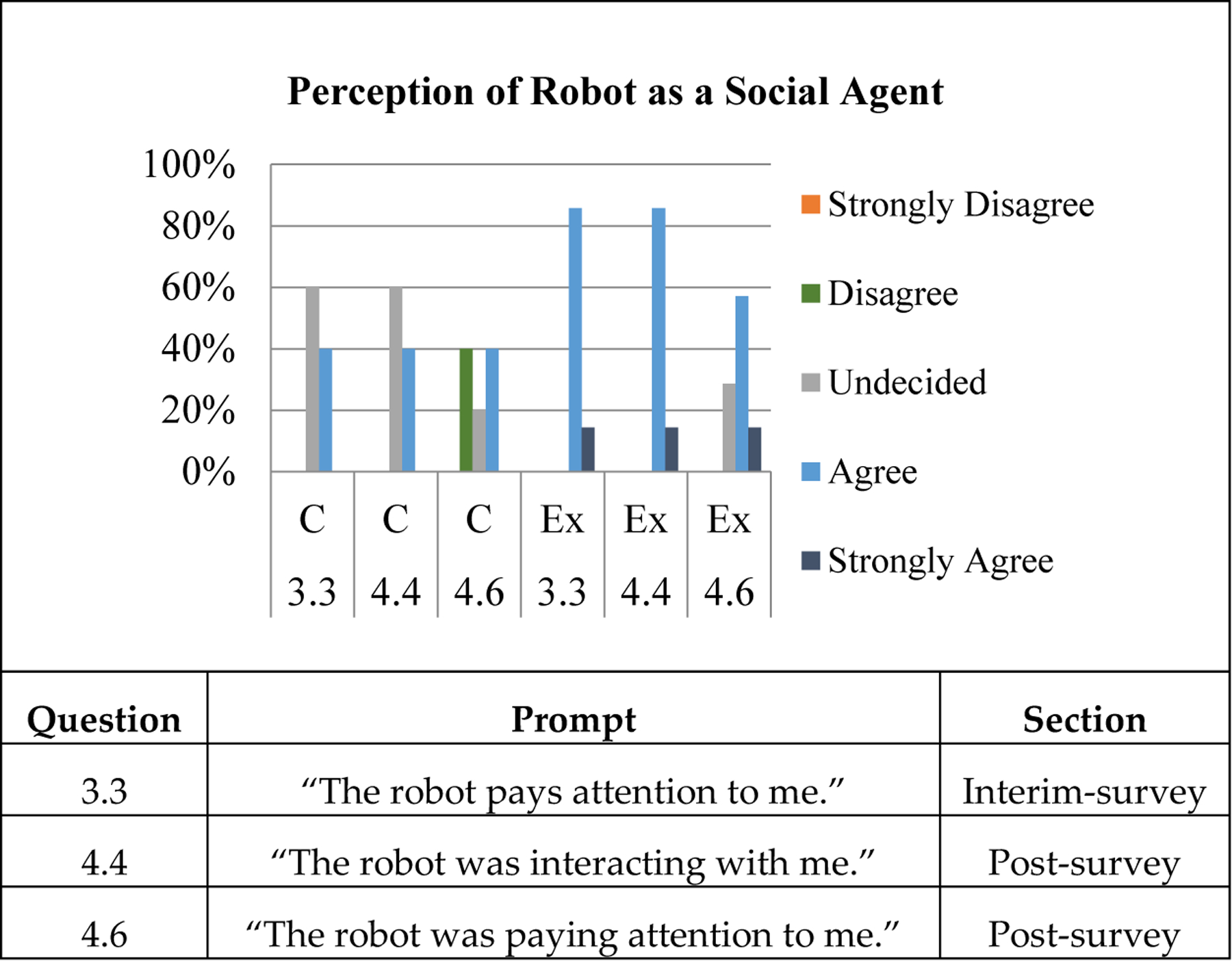
Highlights from the user-survey, overall group impressions. Questions focused on robot believability, before and after completion of the emotion guessing game and imitation sequences.

The full prompts can be seen below, along with user responses, in Fig. 9. The entire survey, as well as all participants’ individual answers, can be found in Appendix B. As a group, the experimental group started and ended with a higher perception of autonomy than the control group did. Although both groups gave repeat rankings for Q3.3 and Q4.4, the experimental group overall responded more positively to Q4.6, “The robot paid attention to me”, than the control group. On an individual level, experimental group participants that saw the imitation move also had a more positive impression of the robot’s autonomy than control participants did.

An unpaired t-test was performed on the results of each of the three survey questions. These can be found in Table 7. The results of the interim-survey question 3.3, “The robot pays attention to me”, was found to have a t-value of −1.208 and a p-value of 0.1275. There was no statistical significance between the answers of both groups for this question. The results of post-survey question 4.4, “The robot was interacting with me”, had a t-value of −2.797 and a p-value of 0.0094. This means that there was a significant difference in the survey responses between groups for this question. Finally, question 4.6, “The robot was paying attention to me” in the post-survey section had a t-value of −1.767 and a p-value of 0.0538. While the difference between answers for this question was technically found not significant, it was very close to the significance threshold of 0.05.

In summary, the experimental and control groups did not significantly differ in opinion (Q3.3) during the interim-survey, which was pre-imitation. However, post-imitation, the two groups had one survey question where their difference in opinion about robot autonomy was nearly significant (Q4.6), and one that was clearly significantly (Q4.4).

## 4. Discussion

The aim of this research was to determine whether human-robot interaction could be improved by including imitation into the robot’s behavior. The hypothesis for this study was that incorporating the use of an imitated move during OP2’s emotion game will cause a change in the participant’s emotional state towards the robot. The dependent variables which measured this hypothesis were participant posture, facial emotional state, incidence of mood contagion, and participant perception of robot autonomy. We examined whether these four approaches individually supported or did not support the hypothesis.

The posture analysis category did not support the hypothesis, as the results of both the experimental and control groups were statistically insignificant. There was no obvious difference in behavior in terms of participant posture whether the robot imitated them or not.

All experimental participants displayed positive facial emotional states towards new emotion primitives at a constant or increased rate after imitation. 40% of control participants displayed a diminished positive emoting rate after the same time checkpoint. Participants smiled more when interacting with OP2 after the robot imitated them. From this, we can infer the experimental group participants shifted to a more positive emotional state and became more engaged with OP2 after imitation. For these reasons, this category supports the hypothesis.

More experimental group participants exhibited mood contagion than control participants. While the control participants only imitated two high arousal emotions (anger and surprise), experimental participants mimicked a wider spectrum of 6 emotions and an imitation move.

The large majority of control participants’ (80%) perception of the robot’s autonomy decreased. Over 70% experimental participants’ perception stayed constant or improved. We can infer that incorporating imitation improved the experimental participants’ perception of the robot as a viable social agent while it interacted with them. These results support the hypothesis.

Three of the four approaches used supported the hypothesis that OP2 performing imitation affects the emotional state of the participant towards the robot. This study shows that, while robotic imitation may not cause the participants to sit in a more open posture, the imitation does encourage the participants to assume a more positive emotional state, to develop a stronger sense of empathy for the robot, and to increase their perception of the robot as an independent social agent.

As an interesting side note, there appeared to be an increased rate of responsive verbalization in experimental participants compared to their control group counterparts. All participants that started out by verbally responding to the robot’s introductory greeting (“Hi! I’m OP2!”) later replied goodbye to it at the end of the session. Experimental participants who did not greet the robot initially were more likely to reply goodbye to it at the end, compared to control participants who did not initially greet the robot. However, due to the small sample size, this observation cannot yet be confirmed with certainty. We are recruiting more participants to solidly confirm our results.

For future works, in order to collect the most accurate data, expansions could be made to the motion learning system. We plan to add filtering to the DMP with GMR algorithm so that it can detect and automatically remove the joint jump errors caused by the Kinect, to prevent the system from crashing.

Future tracking of mood contagion data should be done by running facial emotion detection software, perhaps via a web camera and on the same computer controlling the robot, alongside the Kinect. This would allow for mood contagion to be detected with greater precision, and to identify if certain emotion primitives displayed by OP2 elicited stronger, prolonged or unusual mood responses.

We plan to add additional audio recordings and primitives to OP2, such as “Yes”, “No”, and encouragement to “Guess again”. This way, OP2 would be able to notify the participant whether they had correctly identified the emotion primitive displayed. This would allow the emotion game to be run in a “Wizard of Oz” style, where the experiment could be completed in its entirety without the researcher present at the table. By requiring the participant to talk directly to OP2, the robot may be able to build stronger rapport, thus building a stronger social setting for imitation to take place.

## 5. Conclusions

If robotic imitation can build empathy, trust, and interest in human participants, robots could be used for a host of medical care applications. Robots could provide children with autism a safe and realistic platform to practice social and behavioral situations. Robots could serve as companions to elderly patients, especially those that are bedridden or suffering from illnesses that inhibit their ability for routine social engagement, such as Alzheimer’s or dementia. Finally, robots could offer reliable “supervision” of outpatients. The robot, as a believable social agent, could remind a patient to perform post-surgery exercises. Additionally, the patient could report concerning symptoms to the robot, which could then be transferred on to the doctor. This could reduce the need for in-person checkups – an improvement for patients recovering from an invasive surgery or without easy access to transportation.

## Figures and Tables

**Table 1.** Comparison of human movement to its equivalent performance through OP2. Right side motors (not listed) are equivalent to the left side motors shown.

Angle Name	Composition	OP2 Motor Equivalent
LS_0_	∠(SCyz, SEyz)	M2: L_SHO_PITCH
LS	∠(SCxy, SExy)	M4: L_SHO_ROLL
LE_0_	∠(ESyz, EHyz)	M6: L_ELBOW
LE	∠(ESxy, EHxy)	None

1 Composition abbreviations are identical to those listed in Fig. 3. Composition subscript variables refer to the corresponding three-dimensional planes.

**Table 2.** Comparison of the statistical significance for both groups’ bodily surface area data.

Group	Mean ΔA	Std. Dev	ΔStd. Error Mean	n	DoF	T-Value	P-Value
Control	84.42	111.42	32.16	5	4	1.694	0.165515
Experimental	−16.37	81.62	23.56	7	6	−0.53	0.615143
Experimental (No outlier)	9.47	48.84	19.94	6	5	0.475	0.654811

**Table 3.** Comparison of the statistical significance for both groups’ bodily surface area data.

Group	n	Mean ΔPE	∑ΔPE	∑ΔPE^2^	Std. Dev	Std. Error	DoF	T-Value	P-Value
Control	5	−2.00%	−10	6950	41.62	18.61	9	−0.77	0.46
Experimental	6	13.75%	82.5	4381.25	25.48	10.40			

1 ΔPE refers to the overall change in positive emoting. The control group experiences virtually no change throughout the Emotion Game, while the experimental group has a slight increase in positive emoting after witnessing the imitation move. The before and after emoting rates of individual participants can be found in Appendix A, Table A3.

**Table 4.** Survey prompts focusing on participants’ enjoyment of the Emotion Game.

Question	Prompt	Section
3.4	“I like interacting with the robot.”	Interim-survey
4.1	“The robot’s emotion game was fun.”	Post-survey
4.5	“I like the robot.”	Post-survey

**Table 5.** Participants that displayed mood contagion for specific emotion primitives.

User	Group	Mood Contagion Primitives before Imitation	Mood Contagion Primitives after Imitation
A	control		angry, surprised
C	control		angry
G	experimental	curious	(Data unavailable after curious)
H	experimental	sad, scared, curious	
I	experimental	curious	
J	experimental	imitation move	surprised
K	experimental	disgust, curious	angry

**Table 6.** Summary of survey results focusing on robot autonomy, comparing how individual responses changed after the imitation session.

Perception of Autonomy	Increased	Stayed constant	Decreased
**Control group**	0%	20%	80%
**Experimental group**	43%	28.5%	28.5

**Table 7.** Comparison of statistical significance of autonomy-focused survey questions.

Question	Mean	Mean Experimental	T-Value	P-Value
	Control Group Rating	Group Rating		
3.3	3.6	4	−1.208	0.1275
4.4	3.4	4.14	−2.797	0.0094
4.6	3	3.86	−1.767	0.0538

1 Ratings have been converted from Likert scale wording to a corresponding numerical system. “Strongly Disagree” corresponds with a 1, “Disagree” with a 2, and so forth. The highest possible numerical score is a 5. N value for control group = 5. N value for experimental group = 7.

## Appendix A

**Table A1.** OP2’s emotion primitives, broken down into verbal and physical descriptions.

Emotion	Verbal Prompt	Gesture
Anger	“No – stop it right now!”	Moves head, throws arms up and down like a temper tantrum.
Curiosity	“Hmm, this is pretty interesting.”	Leans forward, mimes scratching its head.
Disgust	“Yuck! That’s gross.”	Turns head away, blocks body with arms.
Embarrassment	“Oh…I feel awkward.”	Looks away, covers face with one arm.
Excitement	“Wow, that is awesome!”	Looks up, throws arms in the air, simulates clapping.
Frustration	“I don’t know what to do!”	Waves arms, then repeatedly taps head with “fingertips”.
Pleased	“Yes! That is perfect.”	Does a “fist pump” gesture – arm raised, then elbow quickly brought back to hip.
Pride	“Good job.”	Looks up, places hands on hips.
Sadness	“I don’t want to talk about it.”	Bends forward, lowers arms.
Fear	“Ahh! Get away from me!”	Crouches down, covers head with arms, shakes head.
Surprise	“Oh my goodness!”	Throws arms up and out, leans back.
Tiredness	Yawning sound.	Turns head to one side, brings one hand up to mouth, stretches other above head.

**Table A2.** Comparison of the change in participant posture, quantified as surface area.

Participant	Group	A_1_	A_2_	ΔA	% Difference
A	Control	51.8	43.2	−8.6	−16.60%
B	Control	90.6	92.4	1.8	+1.99%
C	Control	99.6	130.0	30.4	+30.52%
D	Control	152.7	302.5	149.8	+98.10%
E	Control	45.2	293.9	248.7	+550.22%
F	Experimental	103.6	36.7	−66.9	−64.58%
G	Experimental	85.6	163.0	77.4	+90.42%
H	Experimental	228.4	57.0	−171.4	−75.04%
I	Experimental	102.0	105.5	3.5	+3.43%
J	Experimental	137.5	144.0	6.5	+4.73%
K	Experimental	34.2	77.7	43.5	+127.19%
L	Experimental	119.8	112.6	−7.2	−6.01%

**Table A3.** Change in participant’s emoting behavior towards OP2 before and after imitation.

Participant	Group	Pre-DMP positive emote	Pre-DMP % (n = 5)	Post-DMP positive emote	Post-DMP % (n = 8)	Change in emoting %	Rate of pos. emote per primitive
A	Control	3	60	1	12.5	−47.5	Decreased
B	Control	2	40	1	12.5	−27.5	Decreased
C	Control	2	40	3	37.5	−2.5	Stayed constant
D	Control	1	20	2	25	5	Stayed constant
E	Control	0	0	5	62.5	62.5	Increased
F	Experimental	2	40	6	75	35	Increased
G	Experimental	unavailable	N/A	unavailable	N/A	N/A	Unavailable
H	Experimental	0	0	0	0	0	Stayed constant
I	Experimental	2	40	3	37.5	−2.5	Stayed constant
J	Experimental	1	20	6	75	55	Increased
K	Experimental	1	20	2	25	5	Stayed constant
L	Experimental	3	60	4	50	−10	Stayed constant

## Appendix B: User survey and Results

For each of the questions below, circle the response that best describes how you feel about the statement, where: 1 = Strongly Disagree, 2 = Disagree, 3 = Undecided, 4 = Agree, and 5 = Strongly Agree.

**Table:**

Interim-survey section 1/3					
	Strongly Disagree	Disagree	Undecided	Agree	Strongly Agree
**1. I am proficient in “current” technology (mobile devices, Internet, etc.)**	1	2	3	4	5
**2. I am interested in learning to use new technology.**	1	2	3	4	5
**3. I use electronics and/or computers regularly**	1	2	3	4	5
**4. I am interested in robots.**	1	2	3	4	5
**5. I have prior experience with robots.**	1	2	3	4	5

**Table:**

Interim-survey section 2/3					
	Strongly Disagree	Disagree	Undecided	Agree	Strongly Agree
**1. The robot is friendly.**	1	2	3	4	5
**2. The robot’s voice is distracting.**	1	2	3	4	5
**3. The robot’s voice and choice of words are appropriate.**	1	2	3	4	5
**4. The robot’s movements are natural.**	1	2	3	4	5
**5. I am scared of the robot.**	1	2	3	4	5

**Table:**

Interim-survey section 3/3					
	Strongly Disagree	Disagree	Undecided	Agree	Strongly Agree
**1. I was comfortable performing gestures in front of the robot.**	1	2	3	4	5
**2. Interacting with the robot is boring.**	1	2	3	4	5
**3. The robot pays attention to me.**	1	2	3	4	5
**4. I like interacting with the robot.**	1	2	3	4	5
**5. I would like to play with the robot again.**	1	2	3	4	5

**Table:**

Post-survey section 1/1					
	Strongly Disagree	Disagree	Undecided	Agree	Strongly Agree
**1. The robot’s emotion game was fun.**	1	2	3	4	5
**2. I could understand what emotion the robot was demonstrating.**	1	2	3	4	5
**3. The robot’s movements were not realistic.**	1	2	3	4	5
**4. The robot was interacting with me.**	1	2	3	4	5
**5. I like the robot.**	1	2	3	4	5
**6. The robot was paying attention to me.**	1	2	3	4	5

**Table:**

Interim-survey section 1/3						
Participant	Group	1.1	1.2	1.3	1.4	1.5
A	C	4	4	5	5	4
B	C	5	5	5	4	2
C	C	5	5	5	5	4
D	C	4	4	4	3	2
E	C	5	5	5	5	2
F	E	5	5	5	5	5
G	E	4	5	5	5	4
H	E	5	5	5	5	5
I	E	5	5	5	5	5
J	E	5	5	5	5	4
K	E	5	5	5	5	5
L	E	5	5	5	5	5

**Table:**

Interim-survey section 2/3						
Participant	Group	2.1	2.2	2.3	2.4	2.5
A	C	4	4	5	5	4
B	C	5	5	5	4	2
C	C	5	5	5	5	4
D	C	4	4	4	3	2
E	C	5	5	5	5	2
F	E	5	5	5	5	5
G	E	4	5	5	5	4
H	E	5	5	5	5	5
I	E	5	5	5	5	5
J	E	5	5	5	5	4
K	E	5	5	5	5	5
L	E	5	5	5	5	5

**Table:**

Interim-survey section 3/3						
Participant	Group	3.1	3.2	3.3	3.4	3.5
A	C	4	2	4	4	4
B	C	4	3	3	3	4
C	C	4	1	4	5	5
D	C	4	3	4	4	4
E	C	2	2	3	4	4
F	E	5	2	4	5	5
G	E	4	2	5	5	4
H	E	4	2	4	5	5
I	E	5	1	3	4	4
J	E	3	2	4	4	5
K	E	5	2	4	4	5
L	E	5	1	4	5	5

**Table:**

Post-survey section 1/1							
Participant	Group	4.1	4.2	4.3	4.4	4.5	4.6
A	C	4	4	2	4	5	3
B	C	4	4	4	4	4	2
C	C	5	5	2	3	5	4
D	C	4	4	2	3	4	4
E	C	4	5	2	3	4	2
F	E	5	5	2	4	5	4
G	E	4	5	2	4	5	4
H	E	4	5	4	5	5	4
I	E	4	4	2	4	5	3
J	E	4	4	2	4	4	3
K	E	5	4	2	4	5	4
L	E	5	4	2	4	5	5
